# Multi-site evaluation of Chilean native rhizobacteria for the sustainable management of the walnut root-lesion nematode *Pratylenchus vulnus* (Tylenchida: Pratylenchidae)

**DOI:** 10.3389/fpls.2026.1876371

**Published:** 2026-09-29

**Authors:** Ignacio Vicente-Díez, Javiera Ignacia Yañez, Américo Contreras, José Luis Henríquez-Sáez, Ximena Antiguay, Carlos Castaneda-Alvarez

**Affiliations:** 1 Facultad de Ciencias Agronómicas, Universidad de Chile, Santiago, Chile; 2 Center Agriculture Food Environment (C3A), University of Trento, San Michele all’Adige, Italy; 3 Productos e Insumos Biotecnológicos S.A. (Biogram), Santiago, Chile

**Keywords:** biological control, field validation, *Oerskovia*, PGPR, walnut orchard, walnut root-lesion nematode

## Abstract

*Pratylenchus vulnus* is the most damaging nematode affecting walnut (*Juglans regia* L.) worldwide, but evidence on the performance of biological control agents under commercial orchard conditions remains limited. We evaluated two native Chilean rhizobacteria (*Bacillus mycoides* 25 and *Oerskovia* sp. UCH-1055), previously shown to achieve >98% *in vitro* mortality and 76% biocontrol efficacy under greenhouse conditions, using application strategies combining inoculum concentrations of 10_6_–10_8_ CFU mL^-1^, with or without reinoculation after 30 or 60 days, alongside untreated and chemical controls. Treatments were first evaluated under greenhouse conditions and subsequently validated in multi-site field trials across three commercial walnut orchards representing geographically distinct regions. Greenhouse experiments showed no statistically significant differences among treatments, despite numerical trends favoring bacterial applications. Field trials revealed strong site-dependent efficacy with significant suppression of *P. vulnus* populations in two of three orchards (Buin and Requínoa), corresponding to Reproductive Index reductions of 70–83% relative to untreated controls. Spatial patterns were consistent with reductions in high-density *P. vulnus* areas at these responsive sites. By contrast, rhizobacterial treatments did not produce major consistent changes in the abundance of non-target free-living nematodes. These results provide multi-site field validation of native rhizobacteria against *P. vulnus*, highlighting the biocontrol potential of *Oerskovia* sp. UCH-1055 under responsive field conditions, and the need to evaluate rhizobacterial performance across multiple commercial sites before broader implementation.

## 1 Introduction

Walnut (*Juglans regia* L.) production has expanded steadily worldwide over the last decade, becoming the second most produced tree nut crop globally, with Chile currently ranking among the leading exporters (Bernard et al., 2018). In Chile, walnut orchards cover more than 46,000 ha and represent a major component of the national fruit industry, generating export revenues exceeding US$213.7 million in 2023 and supporting agricultural development across Mediterranean-climate regions (ODEPA, 2023).

The expansion and intensification of walnut cultivation have been accompanied by increasing phytosanitary challenges, among which the root-lesion nematode *Pratylenchus vulnus* (Tylenchida: Pratylenchidae) constitutes a critical constraint (Jones et al., 2013; Gough et al., 2020). As a migratory endoparasite that invades and destroys root cortical tissues, *P. vulnus* is considered the most important nematode affecting walnut worldwide, causing substantial annual losses across major production regions (Castillo and Vovlas, 2007; Dai et al., 2026). Current management relies heavily on chemical nematicides, primarily organophosphates and carbamates such as cadusafos, which face mounting regulatory restrictions - including the global ban on methyl bromide and strict ‘township caps’ on the use of 1,3-dichloropropene. These products frequently exhibit inconsistent field performance, especially in fine-textured soils where fumigants fail to penetrate effectively, while raising serious concerns regarding groundwater contamination and high toxicity to non-target soil organisms (Desaeger et al., 2020; Westphal, 2022). The limited availability of resistant rootstocks further compounds this challenge; even tolerant clonal rootstocks such as ‘VX211’ can suffer damage at low population densities, and the widely used *J. regia* remains highly susceptible (Buzo et al., 2005, 2009). With an economic damage threshold as low as one nematode per 250 cm^3^ of soil and yield losses reaching up to 40%, *P. vulnus* represents a critical, insufficiently addressed threat to sustainable walnut production (Buzo et al., 2009; Gough et al., 2020; Pineda-Escobar et al., 2023; Calsin et al., 2025).

Biological control strategies based on plant growth–promoting rhizobacteria (PGPR) are well established as alternative for the sustainable management of plant-parasitic nematodes (Castaneda-Alvarez and Aballay, 2016; Subedi et al., 2020; Calsin et al., 2025). Numerous studies conducted under controlled conditions have demonstrated that PGPR can interfere with the development and reproduction of both ectoparasitic and endoparasitic nematodes through multiple mechanisms (*e.g.*, direct antagonism, competition within the rhizosphere, and the induction of plant systemic defenses) (Timper, 2014; Hnini and Rabeh, 2025; Vanin et al., 2026). However, their validation under commercial field conditions remains limited, particularly for *P. vulnus* in perennial crops such as walnut (Poveda et al., 2020; Ayaz et al., 2024), which hinders the adoption and deployment of these bio-based tools in commercial agricultural systems (Oka, 2020; Vanin et al., 2026). This field-scale adoption gap is largely associated with strain-specific variability, limited environmental stability, and the difficulty of maintaining bacterial activity under fluctuating soil conditions (Timper, 2014). Successful field implementation requires not only the selection of highly antagonistic strains, but also formulation optimization to improve persistence under abiotic stress and the ability of bacteria to maintain rhizosphere colonization over time (Oka, 2020). In addition, the efficacy of PGPR in commercial settings can be strongly influenced by site-specific factors, including soil properties, temperature fluctuations, moisture regimes, and indigenous microbial communities (Timper, 2014; Poveda et al., 2020). Studies have shown that soil temperatures ranging between 10–15 °C in temperate zones versus 20–25 °C in warmer climates can dramatically influence bacterial establishment and nematicidal activity (Poveda et al., 2020). Such complexity makes biocontrol outcomes difficult to predict and highlights the need for multi-site testing to determine whether PGPR-based products can deliver consistent performance across contrasting production contexts. Ultimately, the scalability of these bio-based tools will depend on defining practical application strategies, including effective inoculum concentrations and reinoculation regimes, that ensure reliable field performance and facilitate their integration into broader integrated pest management (IPM) programs available to growers (Poveda et al., 2020).

We have developed a collection of native rhizobacteria isolated from suppressive soils, with specific strains including *Bacillus mycoides* (formerly *B. weihenstephanensis*) and *Oerskovia* sp. UCH-1055 showing exceptional potential under controlled conditions (Pineda-Escobar et al., 2023). Suppressive soils represent valuable reservoirs of biocontrol candidates because they harbor microbial communities shaped by long-term plant–soil–pathogen interactions, which may favor the selection of antagonistic bacteria with improved rhizosphere competence under local field conditions (Gómez Expósito et al., 2017). These strains achieved *in vitro* mortality rates of 98–100% and biocontrol values of 67–77% under greenhouse conditions, effectively reducing nematode invasion by ~70% while promoting root growth. However, the specific mechanisms underlying these effects remain to be determined, and, as is characteristic of biocontrol development, the critical validation under commercial field conditions remained to be demonstrated.

Given the recognized challenges in translating laboratory success to field applications, this study aimed to evaluate whether the promising greenhouse performance of selected native rhizobacteria translates to effective biocontrol under commercial orchard conditions. We conducted multi-site field validation across three geographically distinct walnut orchards to assess treatment efficacy and determine whether rhizobacterial performance was consistent across field sites. This approach was designed to document site-dependent efficacy under real commercial conditions, rather than to identify the specific environmental drivers underlying such variability, providing applied evidence for the practical evaluation of native rhizobacteria in commercial walnut production.

## 2 Materials and methods

### 2.1 Biological sources and field experiments

#### 2.1.1 Walnut plants

The biological material consisted of English walnut plants (*J. regia* L.) obtained from seeds (nuts) of this cultivar. Nuts were manually opened and the seeds were placed in moist coarse perlite for germination, maintained at 25 °C with 60% relative humidity under complete darkness for one month. Perlite moisture was regularly maintained to ensure adequate hydration and promote uniform seedling emergence. After this period, germinated seedlings were transplanted into individual pots containing a sterilized sand–peat substrate (3:1, v/v) and maintained under greenhouse conditions at 25 °C. Plants were irrigated with distilled water every two days throughout the experiment to maintain adequate moisture conditions.

#### 2.1.2 *Pratylenchus vulnus* source and monoxenic multiplication

Populations of *P. vulnus* were obtained from field soil samples following nematode extraction and species identification. Nematodes were extracted using standard Baermann and Coolen & D’Herde methods (Baermann, 1917; Coolen and D’Herde, 1972). Species identity was confirmed using morphological characters (*e.g*., oval spermatheca and post-uterine sac length, among others) and molecular markers, using the *P. vulnus*-specific primer pair PVUL-F/D3B-R (PVUL-F: 5′-GAAAGTGAACGCATCCGCAA-3′; D3B-R: 5′-TCGGAAGGAACCAGCTACTA-3′), which amplifies a fragment of approximately 287 bp (Al-Banna et al., 2004). To ensure a continuous and contaminant-free supply for bioassays, monoxenic cultures were established using the carrot disc method. Fresh, non-cold-stored carrots (*Daucus carota*) were washed, surface-sterilized in 95% ethanol for 5 min, and flamed under aseptic conditions. Carrots were peeled and cut into 5-mm discs (or cylinders for bottle cultures) and placed on 1% water agar, optionally supplemented with Gamborg medium to promote callus formation. Discs were incubated at 25 °C for 1–4 weeks until callus development. Adult *P. vulnus* were surface-sterilized for 2h in a solution containing streptomycin (2000 ppm) and chlorhexidine (0.001%) and individually transferred onto carrot callus tissue at a 1:4 (male:female) using sterile tools. Cultures were sealed with Parafilm and maintained at 24–25 °C for approximately four months. *Pratylenchus vulnus* were recovered by fragmenting carrot discs and immersing them in sterile tap water for 24 h. The suspension was filtered through a concave metallic sieve, and the extraction process was repeated to maximize nematode recovery.

#### 2.1.3 Bacteria isolations and production

Two native rhizobacteria strains, *B. mycoides* 25 and *Oerskovia* sp. UCH-1055, were utilized (Pineda-Escobar et al., 2023). These strains were previously isolated from suppressive soils and identified via multilocus sequence analysis. For production, the strains were reactivated in 100% Tryptic Soy Broth Agar (TSBA) for 24 hours at 22 °C. Subsequently, they were multiplied in liquid TSB at 20 °C with orbital shaking (180 rpm) until reaching a concentration of 1×10^8^ or 1×10^6^ CFU mL^−1^. These inoculum concentrations were selected based on preliminary assays and previous findings (Pineda-Escobar et al., 2023), which identified these as the most promising concentrations for rhizobacterial biocontrol of *P. vulnus*.

#### 2.1.4 Field site selection and baseline nematode assessment

For the field validation phase, three commercial walnut orchards were selected to specifically assess biocontrol performance across diverse agroecological conditions, representing the variability under commercial orchard conditions, namely in: (a) Hijuelas (32.80° S, 71.17° W; Valparaíso Region), (b) Buin (33.73° S, 70.74° W; Metropolitan Region), and (c) Requínoa (34.28° S, 70.82° W; O’Higgins Region), as illustrated in **Figure 1**. The three orchards were established on predominantly alluvial soils, with textures ranging from loam to sandy loam and good drainage. These sites were identified using the historical database of the Nematology Laboratory at the University of Chile, prioritizing orchards with natural infestations of *P. vulnus* exceeding 100 individuals per 250 cm^3^ of soil. Before the trials began, population levels were verified at each site through composite soil sampling (25 sub-samples per hectare) collected from the tree rows at a depth of 10–25 cm.

**Figure 1:**
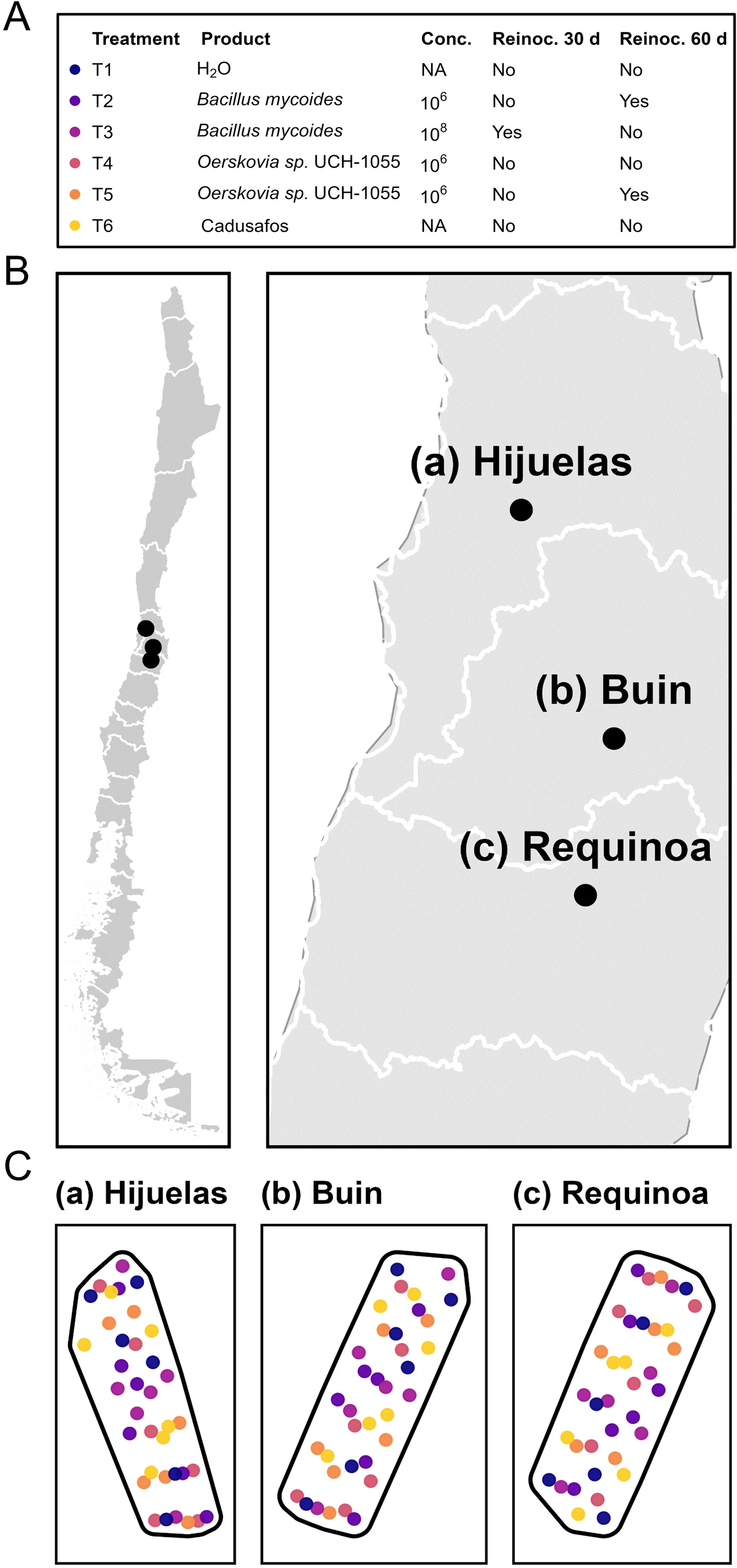
Experimental design and field site distribution for multi-site evaluation of rhizobacterial treatments against *Pratylenchus vulnus*. **(A)** Description of the six treatments applied in greenhouse and field experiments, including product type, inoculum concentration (CFU mL^−1^), and reinoculation regimes at 30 and 60 days, and the color code used to identify each treatment throughout the study. **(B)** Geographic location of the three commercial walnut orchards used for field validation in central Chile: (a) Hijuelas, (b) Buin, and (c) Requínoa. **(C)** Experimental layout within each orchard showing the distribution of treatments across experimental units.

### 2.2 Bioassays

#### 2.2.1 Screening of rhizobacterial treatments against *Pratylenchus vulnus* under greenhouse conditions

A greenhouse bioassay was conducted to evaluate the effect of different rhizobacterial application strategies on plant performance and *P. vulnus* populations. Walnut seedlings grown in 5-L pots were subjected to six treatments, each representing a specific combination of product, inoculum concentration, and reinoculation regime: (T1) water control (H_2_O), (T2) *B. mycoides* 25 at 10^6^ CFU with reinoculation at 60 days, (T3) *B. mycoides* 25 at 10^8^ CFU with reinoculation at 30 days, (T4) *Oerskovia* sp. UCH-1055 at 10^6^ CFU without reinoculation, (T5) *Oerskovia* sp. UCH-1055 at 10^6^ CFU with reinoculation at 60 days, and (T6) the chemical nematicide (cadusafos) (**Figure 1A**). Each treatment was applied to plants at transplanting following the corresponding experimental protocol. Plants were maintained under greenhouse conditions at 25 °C and irrigated according to standard agronomic practices. Each pot was inoculated with *P. vulnus* (2000 individuals) at the beginning of the experiment. Two independent trials were conducted under the same experimental conditions. Plant performance was evaluated at the end of the experimental period by measuring shoot growth expressed as change in plant height (Δ Height, cm) and root weight (g). Nematodes were extracted as described above, including populations from soil and roots. Individuals were counted under a stereomicroscope (ZEISS, Stemi 2000) by trained personnel.

#### 2.2.2 Effect of rhizobacterial treatments on *P. vulnus* and soil nematode communities under field conditions

To bridge the gap between previous controlled-environment results (Pineda-Escobar et al., 2023) and field performance, treatments were first evaluated under greenhouse conditions before multi-site field validation. Field trials were established in three commercial walnut orchards with more than 15 years of cultivation, located in Hijuelas, Buin, and Requínoa, as described before (**Figure 1B**). All of them were naturally infested with *P. vulnus* (>100 individuals per 250 cm^3^ of soil). Within each row, treatments were applied to groups of three adjacent walnut trees, of which only the central tree was sampled and monitored throughout the experiment, constituting the true experimental unit (six sampled trees per treatment per orchard). For each experimental unit, a composite soil sample was formed by pooling eight sub-samples collected around the tree; each composite sample was processed individually for nematode extraction. Consecutive experimental units were separated by two untreated buffer trees (**Figure 1C**). Treatments were applied during the second root growth phase in late summer–early fall, when soil temperatures remain favorable and water stress is reduced, promoting root activity for effective plant–nematode–biocontrol interactions. Nematode populations were assessed at the beginning of the experiment (t = 0) and 90 days after treatment application (t = 90). Soil samples were collected from the rhizosphere of each experimental unit, and nematodes were extracted as described before. Population densities of *P. vulnus*, *Paratylenchus* spp., and free-living nematodes were quantified to evaluate both treatment efficacy and potential effects on the broader soil nematode community. Nematodes were identified and counted under a stereomicroscope by trained personnel, and treatment effects were expressed as Reproductive Index (RI = final population/initial population) values calculated from population densities over time.

### 2.3 Statistical analysis

Greenhouse experiments were analyzed using one-way analysis of variance (ANOVA), with treatment as the fixed factor. The six treatment groups were defined according to product, concentration, and reinoculation regime (T1–T6), as described above. The response variables analyzed were nematode density in roots, the RI (root and soil populations combined), increase in plant height (Δ Height), and dry biomass. Nematode root density and RI were log-transformed as log (x + 1) prior to analysis to improve normality and homogeneity of variance. When the overall treatment effect was significant, mean separation among treatments was performed using Tukey’s honestly significant difference (HSD) test. Differences among treatments are indicated by different letters at *P* < 0.05. Graphical results are presented as means ± SE.

Field data were analyzed separately for each orchard using one-way analysis of variance (ANOVA), with treatment as the fixed factor. The RI was log-transformed using log (x + 1) prior to analysis to improve normality and variance homogeneity. Treatment effect size was estimated using eta-squared (η²). When the overall treatment effect was significant, pairwise comparisons among treatments were conducted using estimated marginal means with Tukey adjustment implemented through the *emmeans* package (P < 0.05). Spatial variation in nematode densities was visualized using interpolation contour maps generated from georeferenced sampling points for each sampling time (t = 0 and t = 90) to assess treatment effects on population hotspots, a critical factor in perennial crop nematode management. These maps were not used to infer statistically significant spatial patterns or treatment effects.

All statistical analyses were conducted using R software (version 4.3.2; R Core Team, Vienna, Austria).

## 3 Results

### 3.1 Greenhouse bioassay: nematicidal effect and effect on plants of the rhizobacteria

Greenhouse evaluation did not discriminate significantly among treatments under controlled conditions (**Figure 2**). No statistically significant differences among treatments were detected for nematode density in roots (F_5,42_ = 0.684, *P* > 0.05) or RI (F_5,42_ = 1.238, *P* > 0.05) (**Figures 2A, B**). Specifically, treatments T3 (*B. mycoides* 25 at 10^8^ CFU mL^−1^ with 30-day reinoculation) and T4 (*Oerskovia* sp. UCH-1055 without reinoculation) showed lower values compared to negative control, although these differences were not statistically significant. Similarly, plant growth parameters showed no significant treatment effects, on plant height increment (Δ Height) or root weight (*P* > 0.05 for both variables) (F_5,42_ = 0.287, *P* > 0.05) (**Figures 2C, D**).

**Figure 2:**
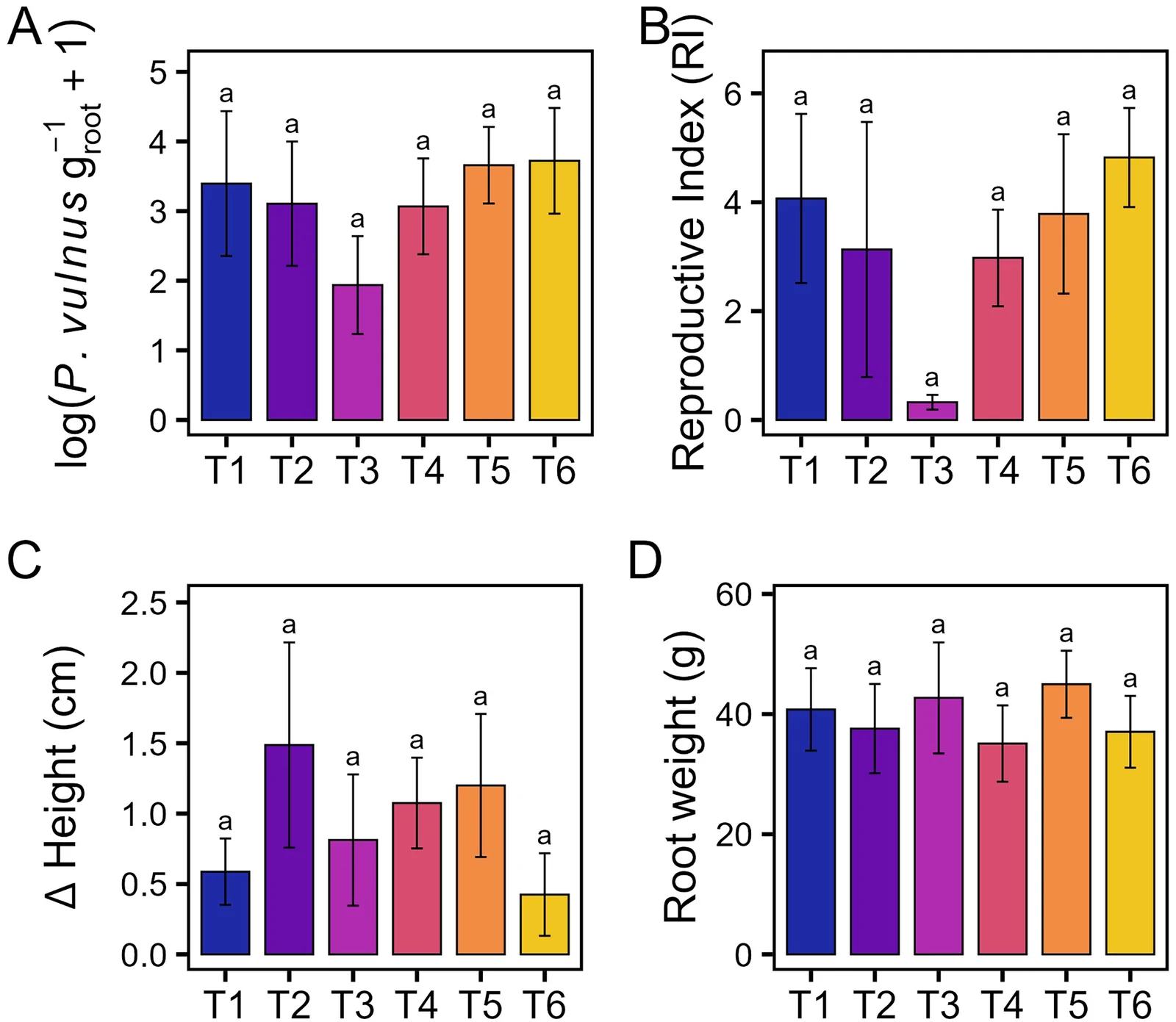
Greenhouse evaluation of rhizobacterial treatments on plant performance and *Pratylenchus vulnus* infestation. Greenhouse bioassay assessing the effect of six treatments (T1–T6) on nematode infection and plant growth parameters. **(A)** Log-transformed nematode density in roots [log(*P. vulnus* g^−1^ root + 1)]. **(B)** Reproductive Index (RI) of *P. vulnus*. **(C)** Increase in plant height (Δ Height, cm). **(D)** Root biomass (g). Bars represent mean ± standard error (SE). Treatments sharing the same letter are not significantly different according to Tukey’s HSD test (P < 0.05).

### 3.2 Field bioassay: nematicidal effect against *Pratylenchus vulnus*

Field trials conducted across three geographically distinct walnut orchards (**Figure 1C**) showed site-dependent biocontrol efficacy. Significant treatment effects were detected in two of three locations (Buin and Requínoa). Spatial distribution maps (**Figure 3**) showed heterogeneous *P. vulnus* distribution and hotspots at the beginning of the experiment (t = 0) in all orchards. After 90 days, a reduction in high-density areas was observed in treated plots, particularly in Buin and Requínoa.

**Figure 3:**
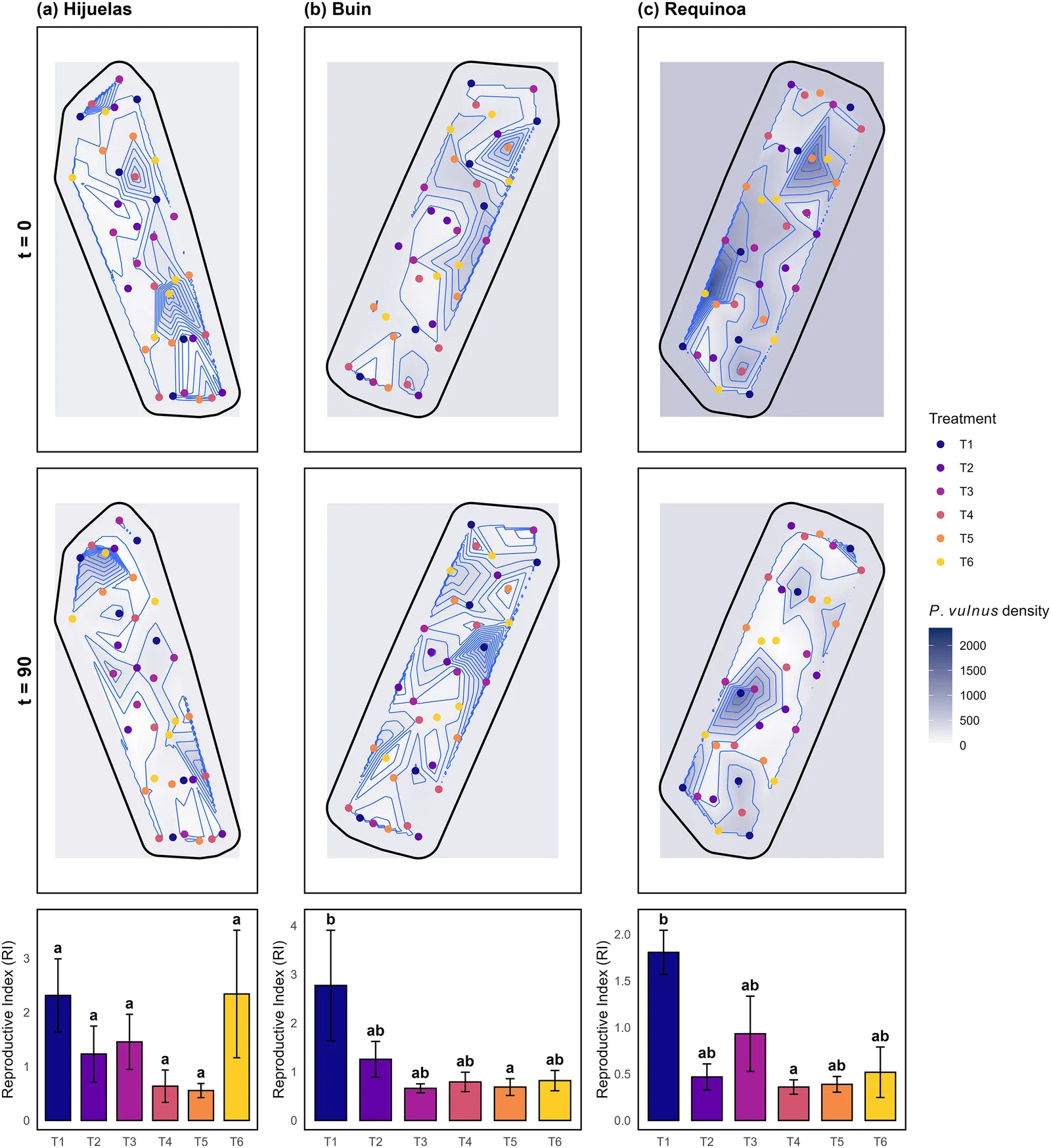
Spatial dynamics and site-dependent effects of rhizobacterial treatments on *Pratylenchus vulnus* populations under field conditions. Top panels correspond to initial sampling (t = 0), and middle panels to sampling after 90 days (t = 90). **(a)** Hijuelas, **(b)** Buin, and **(c)** Requínoa. Points represent sampling locations within each experimental unit, colored according to treatment (T1–T6). Contour maps indicate interpolated nematode density, with darker areas representing higher population densities. Bottom panels show the Reproductive Index (RI) for each treatment and site. Bars represent mean ± SE. Different letters indicate significant differences among treatments (P < 0.05).

Quantitative analysis of RI showed significant site-dependent responses. In Hijuelas, no significant effect of treatment was detected (F_5,30_ = 2.05, *P* > 0.05), although a moderate effect size was observed (η² = 0.25). In contrast, a significant treatment effect was observed in Buin (F_5,30_ = 2.60, P = 0.045; η² = 0.30), with the untreated control exhibiting significantly higher RI values compared to T5 (*Oerskovia* sp. UCH-1055 with reinoculation at 60 d, P = 0.046), achieving approximately 70% reduction in RI. Similarly, in Requínoa, treatments significantly affected RI (F_5,29_ = 2.81, *P* = 0.035; η² = 0.33), with T4 (*Oerskovia* sp. UCH-1055 without reinoculation) differing significantly from the untreated control (*P* < 0.05), resulting in 83% reduction in RI. The remaining treatments showed reduced RI values relative to the control, although none of these pairwise comparisons were statistically significant.

Furthermore, rhizobacterial treatments did not produce consistent or significant alterations in non-target nematode groups across orchards. *Paratylenchus* populations showed variable spatial distributions among locations, with treatment effects differing between sites but without a consistent reduction pattern relative to controls (**Supplementary Figure S1**). Similarly, densities of free-living nematodes remained stable across treatments and sampling times (**Supplementary Figure S2**), with no major changes in abundance detected following rhizobacterial application.

## 4 Discussion

The present study shows the transition of native rhizobacteria from controlled laboratory environments to the ecological complexity of commercial walnut orchards. While greenhouse evaluations yielded non-significant responses, subsequent field validation revealed significant, site-dependent efficacy in two of the three walnut orchards studied. The substantial reductions in the RI observed in Buin (70%) and Requínoa (83%) align with the upper range of efficacy reported for established biological control agents, such as *Bacillus firmus* and *B. velezensis* (Ghahremani et al., 2020; Tian et al., 2022). This field performance suggests that rhizosphere competence may contribute to biocontrol establishment under commercial orchard conditions; however, bacterial persistence and root colonization were not directly measured in the present study, and therefore their role in the observed field responses remains hypothetical (Timper, 2014; Ayaz et al., 2024).

Previous greenhouse studies have reported biocontrol levels of 67–77% against *P. vulnus* using rhizobacterial treatments (Pineda-Escobar et al., 2023). In our study, *B. mycoides* 25 and *Oerskovia* sp. UCH-1055 yielded variable and non-significant responses, despite including a higher inoculum density for B. mycoides 25 (10^8^ CFU mL^−1^, T3). This variability among studies suggests that the nematicidal potential of native rhizobacteria is not merely a function of inoculum concentration, but may also depend on the host’s phenological state, greenhouse conditions, and soil physicochemical properties, such as pH, redox conditions, and ionic composition, which may influence rhizobacterial establishment and biofilm formation, a critical prerequisite for PGPR-mediated antagonism (Ghahremani et al., 2020). Moreover, substrate type and soil physicochemical conditions can directly affect nematode activity by modifying water availability, aeration, oxygen diffusion, and movement through soil pores, while also influencing rhizobacterial establishment and persistence in the rhizosphere (Jones, 1975; Ayaz et al., 2024). Notably, the chemical reference treatment, cadusafos, also had no significant effect on *P. vulnus* populations under our greenhouse conditions, despite being a widely used nematicide with documented efficacy against plant-parasitic nematodes. These results underscore that greenhouse results may not translate to field conditions, highlighting the need for standardized protocols, monitoring, and new hypotheses to explain variability. These findings also raise the question of whether standard greenhouse bioassays, as implemented here, retain sufficient discriminatory power to serve as a reliable pre-selection step before field validation. Future studies may benefit from complementary screening criteria, such as longer exposure periods, additional response variables (*e.g*., root colonization density), or higher replication, to better identify candidates with field-relevant biocontrol potential before committing resources to multi-site trials.

On the other hand, the substantial reductions in the RI achieved in Buin and Requínoa demonstrate that native rhizobacteria, specifically *Oerskovia* sp. UCH-1055, can perform at levels comparable to chemical standards such as cadusafos. These results align with the efficacy ranges reported for high-performing PGPR strains in other perennial crops (Ghahremani et al., 2020; Subedi et al., 2020). However, the lack of significant suppression in Hijuelas, despite a moderate effect size, underscores the site-specific nature of biological control. As noted by Seong et al. (2021), the efficiency of biocontrol agents depends critically on environmental factors such as soil pH, temperature, moisture content, and soil type, all of which affect their survival and field effectiveness. However, baseline physicochemical characterization of the orchard soils was not performed in the present study; therefore, although treatment efficacy was clearly site-dependent, the specific edaphic factors underlying these differences, such as pH, texture, organic matter, or nutrient status, could not be identified. Likewise, Mozgovaya et al. (2002) emphasize that the success of introduced bacteria in the soil environment relies on their ability to transition from spores to active vegetative cells and successfully migrate toward the root zone. Their findings suggest that the colonization of the rhizosphere is a prerequisite for persistence, as the bacteria must interact effectively with indigenous soil organisms to maintain their nematicidal activity *in situ*. The absence of statistical significance in Hijuelas should be interpreted cautiously, as the observed effect size (η² = 0.25) suggests a biologically meaningful treatment effect that may have been masked by high within-site variability or insufficient statistical power at this location. This pattern is consistent with the inherent heterogeneity of nematode spatial distributions in field conditions, where population hotspots can inflate variance and reduce the sensitivity of treatment comparisons. Our findings emphasize that multi-site field evaluations are essential to capture variability in treatment performance across commercial orchards and to generate hypotheses about the environmental factors that may influence biocontrol efficacy.

Finally, given the applied nature of this study and its focus on evaluating rhizobacterial performance across multiple field sites, treatment design inevitably encompassed multiple interacting factors—such as bacterial species, inoculum concentration, and reinoculation regimes—without following a fully factorial approach. Therefore, treatment-specific responses should be interpreted as the performance of combined application strategies rather than as isolated effects of bacterial species, inoculum concentration, or reinoculation frequency. While this strategy allowed us to capture the variability inherent to commercial orchard conditions, it also limited our ability to disentangle the individual contributions of these variables. Future research should aim to isolate and evaluate these factors more explicitly, particularly dose–response relationships, inoculation regimes, formulation parameters, and physicochemical characterization of the soils, in order to better understand the drivers of rhizobacterial efficacy under field conditions. These factors may still play a role in fine-tuning bacterial performance under specific environmental contexts. As a first field approximation, this study evaluated rhizobacterial performance during a single application window, the second root growth phase in late summer–early fall, and future work should assess whether extended or season-long treatment regimes further consolidate the biocontrol outcomes observed here.

## 5 Conclusions

This study provides, to our knowledge, the first multi-site field evidence that native rhizobacteria, particularly *Oerskovia* sp. UCH-1055, can achieve meaningful suppression of *P. vulnus* populations in commercial walnut orchards, with RI reductions comparable to chemical standards at responsive sites. The site-dependent outcomes suggest that rhizosphere competence and local field conditions may influence biocontrol success, although these factors require direct evaluation in future studies. Moving forward, advancing the consistency of rhizobacterial biocontrol will require integrating multi-site validation with optimized inoculation strategies, dose–response characterization, and a deeper mechanistic understanding of rhizosphere colonization under diverse orchard conditions.

## Data Availability

The raw data supporting the conclusions of this article will be made available by the authors, without undue reservation.
