# Supplementary material for: Multi-site evaluation of Chilean native rhizobacteria for the sustainable management of the walnut root-lesion nematode Pratylenchus vulnus (Tylenchida: Pratylenchidae)
Source: Front Plant Sci. 2026 Sep 29;17:1876371. doi: 10.3389/fpls.2026.1876371 (PMC13658139; doi:10.3389/fpls.2026.1876371)
Supplement: Supplementary file 1 [file Supplementaryfile1.pdf]

Supplementary Material

Multi-site evaluation of Chilean native rhizobacteria for the sustainable management of the walnut lesion nematode *Pratylenchus vulnus* (Tylenchida: Pratylenchidae)

Vicente-Díez I., Yañez J.I., Contreras A., Henríquez-Sáez J.L., Antiguay X., Castaneda-Alvarez C.

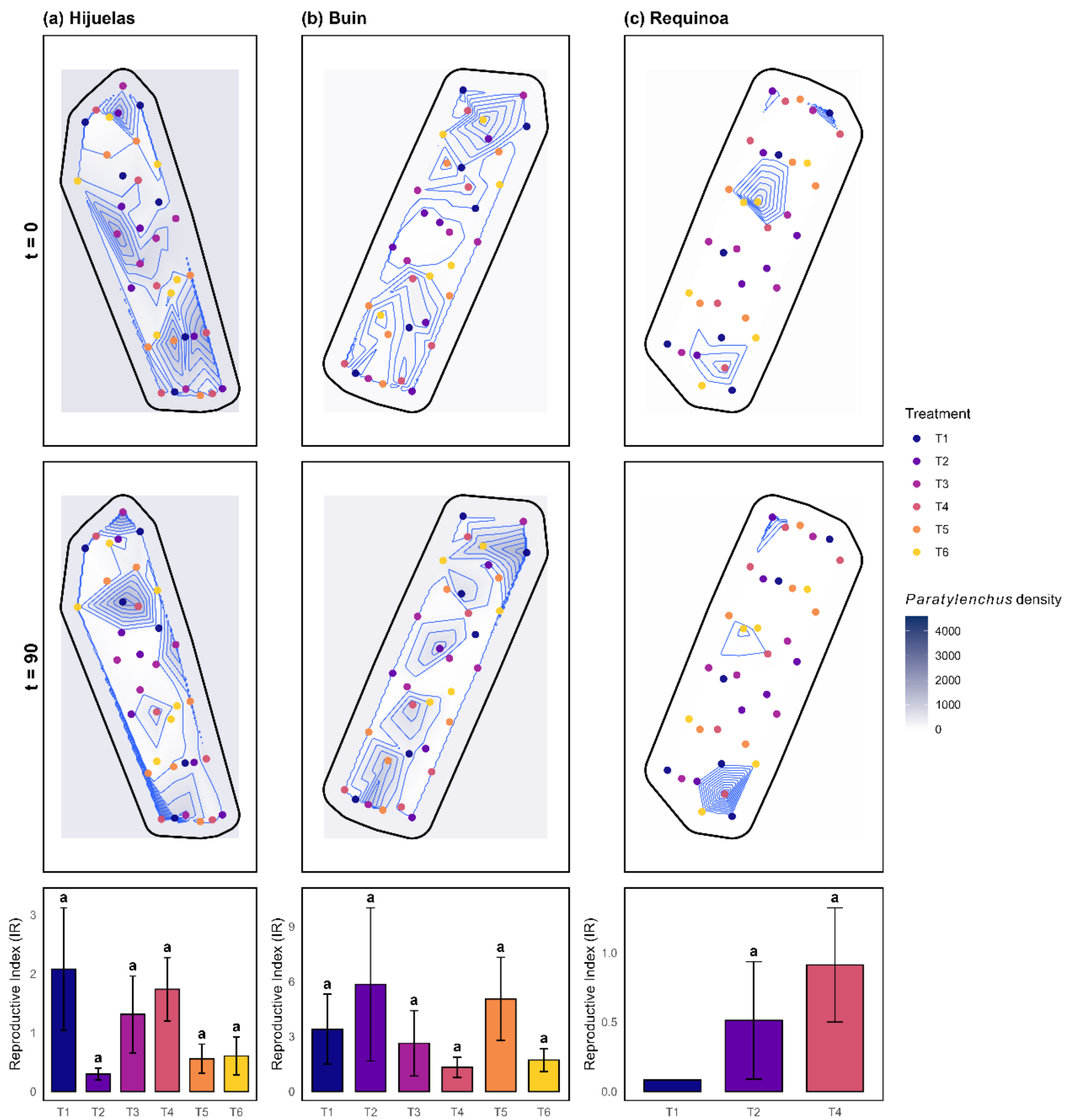

Figure S1. Spatial dynamics and site-dependent effects of rhizobacterial treatments on *Paratylenchus* spp. populations under field conditions. Top panels correspond to initial sampling (t = 0), and middle panels to sampling after 90 days (t = 90). (a) Hijuelas, (b) Buin, and (c) Requinoa. Points represent sampling locations within each experimental unit, colored according to treatment (T1–T6). Contour maps indicate interpolated nematode density, with darker areas representing higher population densities. Bottom panels show the reproductive index (RI) for each treatment and site. Bars represent mean ± SE.

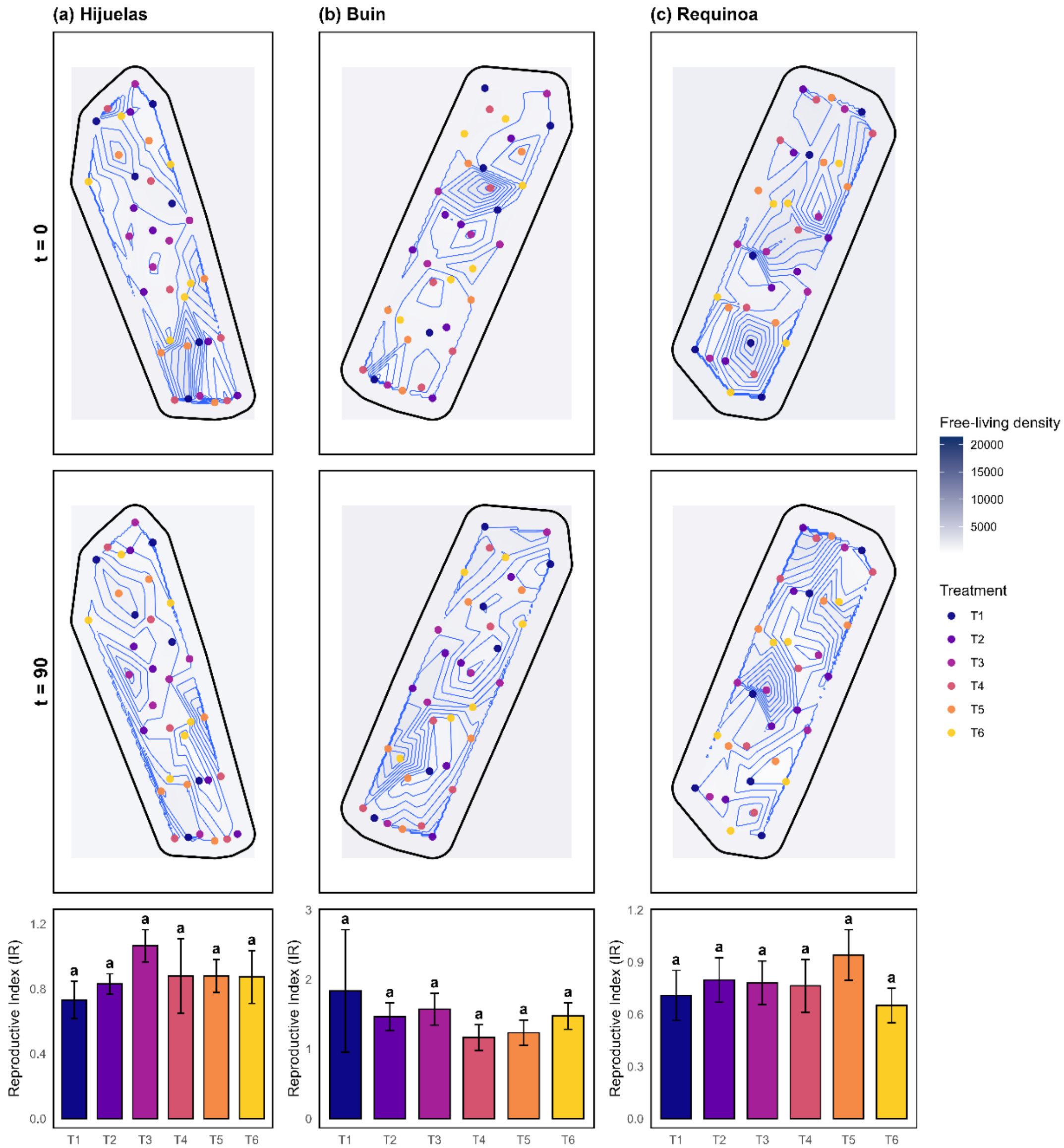

Figure S2. Spatial dynamics and site-dependent effects of rhizobacterial treatments on free-living nematode populations under field conditions. Top panels correspond to initial sampling (t = 0), and middle panels to sampling after 90 days (t = 90). (a) Hijuelas, (b) Buin, and (c) Requinoa. Points represent sampling locations within each experimental unit, colored according to treatment (T1–T6). Contour maps indicate interpolated nematode density, with darker areas representing higher population densities. Bottom panels show the reproductive index (RI) for each treatment and site. Bars represent mean ± SE.
